# A deep convolutional neural network for segmentation of whole-slide pathology images identifies novel tumour cell-perivascular niche interactions that are associated with poor survival in glioblastoma

**DOI:** 10.1038/s41416-021-01394-x

**Published:** 2021-04-29

**Authors:** Amin Zadeh Shirazi, Mark D. McDonnell, Eric Fornaciari, Narjes Sadat Bagherian, Kaitlin G. Scheer, Michael S. Samuel, Mahdi Yaghoobi, Rebecca J. Ormsby, Santosh Poonnoose, Damon J. Tumes, Guillermo A. Gomez

**Affiliations:** 1 Centre for Cancer Biology, SA Pathology and University of South Australia, Adelaide, SA Australia; 2 Computational Learning Systems Laboratory, UniSA STEM, University of South Australia, Mawson Lakes, SA Australia; 3 Department of Mathematics of Computation, University of California, Los Angeles (UCLA), CA USA; 4 Mashhad University of Medical Sciences, Mashhad, Iran; 5 Adelaide Medical School, University of Adelaide, Adelaide, SA Australia; 6 Electrical and Computer Engineering Department, Department of Artificial Intelligence, Islamic Azad University, Mashhad Branch, Mashhad, Iran; 7 Flinders Health and Medical Research Institute, College of Medicine & Public Health, Flinders University, Adelaide, SA Australia; 8 Department of Neurosurgery, Flinders Medical Centre, Bedford Park, SA Australia

**Keywords:** CNS cancer, Cancer microenvironment, Machine learning

## Abstract

**Background:**

Glioblastoma is the most aggressive type of brain cancer with high-levels of intra- and inter-tumour heterogeneity that contribute to its rapid growth and invasion within the brain. However, a spatial characterisation of gene signatures and the cell types expressing these in different tumour locations is still lacking.

**Methods:**

We have used a deep convolutional neural network (DCNN) as a semantic segmentation model to segment seven different tumour regions including leading edge (LE), infiltrating tumour (IT), cellular tumour (CT), cellular tumour microvascular proliferation (CTmvp), cellular tumour pseudopalisading region around necrosis (CTpan), cellular tumour perinecrotic zones (CTpnz) and cellular tumour necrosis (CTne) in digitised glioblastoma histopathological slides from The Cancer Genome Atlas (TCGA). Correlation analysis between segmentation results from tumour images together with matched RNA expression data was performed to identify genetic signatures that are specific to different tumour regions.

**Results:**

We found that spatially resolved gene signatures were strongly correlated with survival in patients with defined genetic mutations. Further in silico cell ontology analysis along with single-cell RNA sequencing data from resected glioblastoma tissue samples showed that these tumour regions had different gene signatures, whose expression was driven by different cell types in the regional tumour microenvironment. Our results further pointed to a key role for interactions between microglia/pericytes/monocytes and tumour cells that occur in the IT and CTmvp regions, which may contribute to poor patient survival.

**Conclusions:**

This work identified key histopathological features that correlate with patient survival and detected spatially associated genetic signatures that contribute to tumour-stroma interactions and which should be investigated as new targets in glioblastoma. The source codes and datasets used are available in GitHub: https://github.com/amin20/GBM_WSSM.

## Introduction

Glioblastoma is the most frequently diagnosed and aggressive type of brain cancer, accounting for 80% of primary malignant brain tumours of the central nervous system (CNS), and 60% of all malignant brain tumours in adults.^1^ There are ~100,000 new cases of glioblastoma diagnosed each year worldwide,^1,2^ with a 1.6-fold higher prevalence in men.^3^ While rare relative to overall cancer incidence, glioblastoma accounts for 2.5% of total cancer-related deaths.^1^

The clinical management of glioblastoma clinical management has not improved in the last 30 years.^4^ First-line therapy for newly diagnosed glioblastoma is maximal safe resection of the tumour, followed by concurrent chemo-radiation and maintenance chemotherapy.^5^ Despite these aggressive treatments, the disease almost inevitably recurs, in which case there is no standard treatment available.^6^ This lack of progress could be attributed to two possible reasons (i) extensive intra- and inter-tumour heterogeneity and (ii) the highly invasive and infiltrative nature of these tumours, both of which are dependent on the interactions of the tumour cells with the surrounding microenvironment.^7^

Gliomas are graded and categorised according to the World Health Organization (WHO) guidelines based on a combination of histologic and molecular features.^8^ Grade IV gliomas correspond to glioblastoma and essential diagnostic features include atypical glial cells, brisk mitotic activity, evidence of microvascular proliferation (MVP) and/or significant necrosis. MVP typically appears as glomeruloid tufts of multi-layered endothelial cells that are mitotically active along with smooth muscle cells or pericytes. Necrosis is a fundamental feature of glioblastoma and, together with the presence of blood vessels, is the strongest predictor of aggressiveness.^9–11^

Although analysis of gene-expression data (bulk RNA-seq) available from The Cancer Genome Atlas (TCGA) has enabled the successful identification of the molecular signatures/biomarkers associated with the different GBM tumour subtypes, to date, GBM treatment based on this information alone has not resulted in improved patient survival.^4,12^ GBM treatment is further complicated by the fact that glioblastoma tumours also exhibit a high level of cancer cell heterogeneity and plasticity.^13–19^ Indeed, tumour plasticity and the capacity of cancer stem cells to partially and/or reversibly differentiate into different cancer cell populations is believed to be the main cause of resistance to therapy and the development of tumour recurrence.^13,16,20^ Understanding the mechanisms that contribute to glioblastoma plasticity and how it is shaped by the tumour microenvironment (i.e. stem cell niches) has been difficult. This is partly due to the lack of appropriate tools to unravel the spatial and functional interactions that occur between tumour cells and cells in the tumour niche, and to predict which interactions are important regulators of cancer stem cell plasticity and the capacity of cancer cells to infiltrate the surrounding healthy brain tissue.^12^

Recently, a Decision Forests statistical machine learning-driven algorithm (“Mill”) was used by the Ivy GAP to identify and segment tumour regions and label the anatomic features in ~12,000 histological images, which were then laser-capture micro-dissected and subjected to bulk RNA-seq.^21^ The Ivy GAP data constituted a significant advance in the field and has allowed the identification of signatures associated with different tumour regions.^21^ However, it has limitations, as the results were highly dependent on the segmentation algorithms that were implemented for the segmentation of the different tumour regions^22–25^ and inherently biased due to the small number (*n* = 32) of patient samples analysed.^21^ Furthermore, although multiple sections/regions were processed for data augmentation and the creation of the Ivy GAP database, the small number of patients recruited to the project did not permit the identification of genetic signatures or key demographic characteristics associated with survival and/or particular molecular profiles of the tumours.^21^ Moreover, the gain in insight into spatial resolution by attributing gene signatures to specific tumour regions was not translated into an understanding of the expression of these signatures by specific cell types, as limited cellular ontology analysis was performed on this data, due to the limited number of datasets and bioinformatic tools at the moment of its publication. Consequently, there is currently no available data that permits the identification of the cell populations that are present in each of these tumour anatomic locations and the corresponding transcriptional programs that underpin tumour-stroma interactions that may contribute to poor survival. Gaining insight into these aspects would be critical to progress our understanding on the biology of these very aggressive brain tumours.^12^

Here, we implemented a deep convolutional neural network (DCNN) model for semantic segmentation of histopathological (Haematoxylin-Eosin, H&E) images trained on the Ivy GAP dataset to improve the quality of segmentation of different brain tumour regions (Supplementary Fig. 1A). We sought a pathologist’s opinion to advise on the removal of outliers (Supplementary Fig. 1B includes some examples) in the input dataset and for verification of results, that together contribute to improve the accuracy of segmentation of different brain tumour regions. The resulting model was then employed to segment TCGA histopathological images and correlation analysis between gene expression and tumour region size was used for the identification of different gene signatures associated with different tumour regions as well as the different cell types that express them. We further combined these results with single-cell RNA sequencing (scRNA-seq) data to investigate tumour-stroma interactions that contribute to poor prognosis in glioblastoma.

## Methods

### Histology image datasets

We used the Ivy GAP dataset, as the main dataset for training our DCNN semantic segmentation model (GBM_WSSM). This dataset includes 32 patients diagnosed by primary surgery type with a total of 805 whole-slide images (WSIs) along with their corresponding ground truths.^21^ Three distinct datasets (training, validation and test) were created by randomly splitting the data in the ratio of 14:1:1. Twenty eight patients with 687 WSIs were allocated to the training dataset, two patients with 52 WSIs were allocated to the validating dataset and two patients with 48 WSIs were allocated to the testing dataset. The validation set was used to evaluate the performance of our models during the training phase while the test set includes unseen samples for checking the model after training. An additional dataset was downloaded from TCGA^26^ and includes 640 whole-slides H&E histopathological images from 329 glioblastoma tumours. The GBM_WSSM was applied to this dataset to produce the masks corresponding to all patients. Those masks were then used for further biological investigation as described in the paper.

### TCGA RNA-Seq dataset

The following filters were applied to extract RNA glioma datasets from TCGA for correlation analysis. Project: TCGA GBM, Disease Type: gliomas, Vital Status: dead; Data Category, transcriptome profiling; Data Type: RNA-Seq. The resulting cases were further filtered by the availability of matched and successfully segmented whole-slide images from the same tissue portion used for RNA-seq analysis (Supplementary Table 1). FPKM-UQ data from this cohort of patients was used and when available, replicates measurements from the same patient were averaged. ENSG to gene name conversion was performed using the Biomart tool from Ensembl (https://m.ensembl.org/biomart/martview/e5476740b357b1b968ca8507fd4d853e) using Homo sapiens (human) genome assembly GRCh38.p13 from Genome Reference Consortium.

### Image pre-processing

In the pre-processing stage, all WSIs and GTs from the Ivy GAP dataset were first visually checked by the trained pathologist of our team (N.S.B.) and 18 of 805 were removed as outliers (Supplementary Fig. 1B) because of high-level noise presenting poor segmentation in the masks. All images and masks were then re-sized to four different scales: 4096 × 4096, 2048 × 2048, 1024 × 1024 and 512 × 512 from the original size 18,000 × 15,000. Afterwards, different sizes of patches (1024 × 1024, 512 × 512, 256 × 256) were systematically extracted (not randomly) from the re-sized images and their masks. Therefore, the extracted patches were the entire datasets obtained from images and used in the training phase. Patches were then normalised by using a pixel-normalisation method into the domain [0,1] by dividing all pixel values by the largest pixel value among all patches i.e. 255. For data augmentation purposes, random crops and vertical flips were applied in the training phase to all models.

### Algorithms

**Fig. 1:**
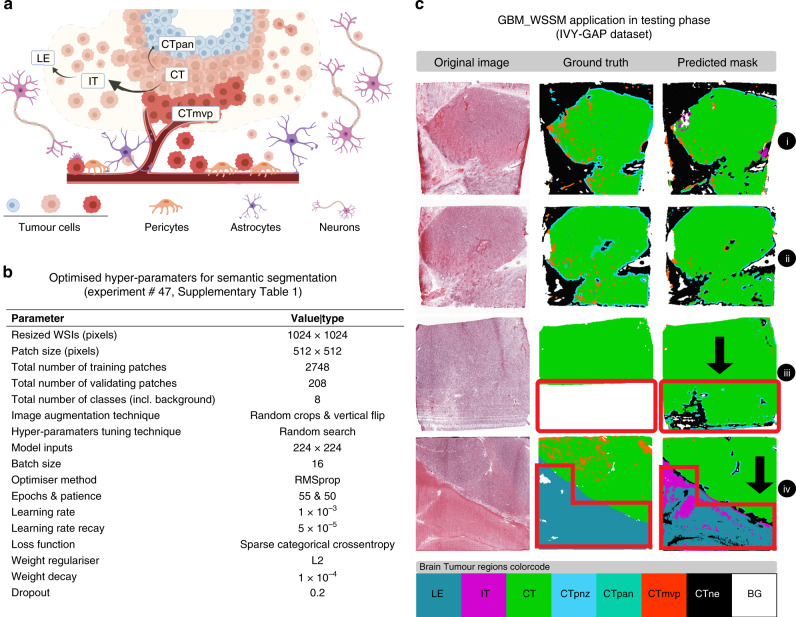
The training and testing phases of the GBM_WSSM model on the IVY-GAP dataset. **a** Schematic representation of different brain tumour regions in glioblastoma. **b** Optimal experiment details for GBM WSI semantic segmentation model (GBM_WSSM). **c** Representative images from segmentation results of the proposed model in the testing phase (GBM_WSSM segmentation superiority has been shown by black arrows).

For semantic segmentation of the eight different brain tumour regions inside the GBM WSIs, a customised fully convolutional DenseNets for semantic segmentation with 103 layers (based on the tiramisu design)^23^ was trained and validated on the normalised patches. These regions include (Fig. 1a) leading edge (LE), infiltrating tumour (IT), cellular tumour (CT), cellular tumour microvascular proliferation (CTmvp), cellular tumour pseudopalisading region around necrosis (CTpan), cellular tumour perinecrotic zones (CTpnz), cellular tumour necrosis (CTne) and background (BG, i.e. area void of tissue). Spearman correlation analysis (Matlab 2019b, see also Supplementary Materials 1, 2 and 3) between gene expression and brain tumour region size was used to identify genetic signatures that are specific to each tumour region (Supplementary Table 2). *p*-values against the null hypothesis that there is no correlation between these variables were calculated for each Spearman correlation test and are also included in Supplementary Table 2. These *p*-values were not corrected for multiple comparisons^27–30^ since (i) the *p*-value distribution is not the same across all brain tumour regions and is not always uniformly distributed (Supplementary Fig. 2, A and B), and (ii) there are significant correlations within each of the variables (i.e. genes vs genes expression; region size vs region size) used for Spearman calculations (Supplementary Fig. 2, C), which suggest that multiple Spearman tests were not fully independent of each other.

### Performance evaluation

We trained 49 different networks and used the random search approach for hyperparameter tuning in each one (Supplementary Table 3). We used sparse categorical cross-entropy, and RMSprop, as loss function and optimiser method, respectively. The number of epochs in different experiments varied between 30 and 150. For prevention of overfitting, dropout technique and L2 weight regulariser were applied. Experiments’ performance comparison was performed based on the evaluator used in the original paper^23^ and by using pixel-wise classification accuracy, where each predicted pixel in the mask produced by a DCNN model is compared with the corresponding pixel in the original ground truth.

### Hardware and software

We used python as the programming language, Keras,^31^ a high-level neural networks API with the Tensorflow platform,^32^ and trained all networks using four NVIDIA 1080Ti GPUs. PRISM and Matlab2019b were used for Pearson’s and Spearman correlation analysis (codes provided Supplementary Materials 1, 2 and 3). Survival plots were generated in Matlab using MatSurv function,^33^ Cell ontology analysis was performed using CellKb (https://www.CellKb.com.^34^), Clustergrams were generated using Morpheus (https://software.broadinstitute.org/morpheus) and Matlab2018 (Supplementary Materials 1). Euclidean distance was used for clustering of variables. Venn diagrams were drawn using InteractiVenn.^35^ Gene ontology analysis was performed using Gorilla^36^ and network graphs generated using Cytoscape.^37^ Scientific Illustrations were created with BioRender.com and all Figures were compiled using Adobe Illustrator 2020.

### scRNA-seq experiments and data analysis

Preparation of samples, library preparation and bioinformatic pipelines for the generation of curated scRNA-seq data from three resected glioblastoma tissue samples was described before.^38^ Cell quality control and clustering were done using Seurat version 3.0^39^ as we described before^38^ and analysis of paracrine ligand–receptor pairs between clusters were done using SingleCellSignalR.^40^

We use average expression per cluster of differential expressed genes in this data for analysis of cell ontology and hierarchical clustering analysis.

## Results

### A DCNN model improves semantic segmentation accuracy of whole-slides histopathological images of glioblastoma

Large labelled datasets are essential parts of the training phase of the deep learning models with supervised learning. Sometimes, it can be seen that the labels (ground truths) for training deep learning models have been generated by non-experts or automated methods and hence, the level of noise in such labels is typically higher than the labels annotated by experts. However, recent studies confirm that DCNNs are extremely robust to handle the high level of noisy labels in supervised learning approaches since well-designed DCNNs applied to sufficiently large and diverse dataset do not memorise the data and they learn the dominant patterns shared among all samples.^41^

The Ivy GAP portal (http://glioblastoma.alleninstitute.org/) is a freely accessible online database that contains GBM slides and their corresponding ground truths annotated using a statistical machine learning method. Although this dataset is very helpful for further research, we identified variable segmentation accuracy in some masks by manual inspection.

Because of the wide use of the Ivy GAP histopathological images database within the brain cancer research community, we decided to use the GBM slides and their corresponding labels from the Ivy GAP to train a deep convolutional neural network (DCNN) architecture and obtain a semantic segmentation model of glioblastoma histopathological images. To increase the accuracy of the semantic segmentation model, we took the advantage of DCNN models in addressing noisy labels^41^ and introduced the pathologist opinion after the training phase of each experiment (Supplementary Fig. 1, Supplementary Table 3). Then, the best model was applied to TCGA as a larger database containing more GBM patients to segment its GBM slides.

Our results indicated that WSI and mask re-sizing from its original size 18,000 × 15,000 to 1024 × 1024 and extracted patches with the size of 512 × 512 led to the highest segmentation accuracy, reaching ~70% determined by pixel by pixel comparison (model 47 in Supplementary Table 3, and Fig. 1b). However, when compared to the original H&E image, the model accuracy was better than the ground truth (as can be seen in Fig. 1c). In this figure, it can be seen that the segmentation accuracy in our model is better than that achieved in the original GTs, particularly in the third and fourth examples shown by the black arrows and the red polygons. Based on our pathologist’s advice, our implemented DCNN model in segmentation of GBM slides has the following advantages (in comparison with the original GTs produced by a statistical machine learning model):

- The DCNN model can segment unclear regions in the original slide (Fig. 1c, iii)
- The DCNN model is able to properly detect the regions corresponding to the Infiltrating Tumour (specified by the purple colour) and Cellular Tumour Necrosis (specified by the black colour) regions without over-segmenting the Leading-Edge region (Fig. 1c, iv)
- The DCNN model can tackle the problem of over-segmentation of the Cellular Tumour Microvascular Proliferation region (specified by the orange colour in the masks, Fig. 1c, i, ii, and iv)
- The DCNN model can tackle the problem of over-segmentation of the Cellular Tumour (specified by the green colour) region (in almost the majority of the masks produced).

We reported the results using the global accuracy metric across all eight classes^23^:

$${\mathrm{Accuracy}} = \frac{{{\mathrm{TP}} + {\mathrm{TN}}}}{{{\mathrm{TP}} + {\mathrm{TN}} + {\mathrm{FP}} + {\mathrm{FN}}}}$$

This equation calculates the ratio of the correctly classified pixels (TP + TN) with regard to the total pixels (TP + TN + FP + FN). We have used the accuracy metric as an evaluator to compare between our 49 different DCNN experiments during the training phase, hyper-parameters tuning, and to select the best models. For this purpose, we applied the Human_In_The_Loop (HITL) approach, which is a setting in a loop where an expert can insert prior knowledge into an AI machine to enhance its output.^42^ In our case, the HITL approach entailed our pathologist supervising/evaluating the results (segmented masks) obtained by the experiments with the better performance (i.e. higher accuracy, see also Supplementary Fig. 1). Subsequently, if the results were not satisfactory (based on the pathologist’s opinion), even if the accuracy values achieved by the experiment was good, the experiments were repeated by adjusting the hyper-parameters to eventually achieve a better segmentation result among all regions than the previous model producing the original GTs. Furthermore, while we have eight distinct regions/classes, we do not see a significant class imbalance in the original GTs, particularly in the main parts i.e. Cellular Tumour, Infiltrating Tumour, Leading Edge and Necrosis. The Background (BG) region also covers a small portion of each GT and the ~70% segmentation accuracy has been achieved among all eight classes. For simplicity, we call our best model “GBM_WSSM”.

### Different brain tumour regions are associated with distinct GBM mutation profiles that are indicative for poor prognosis

**Fig. 2:**
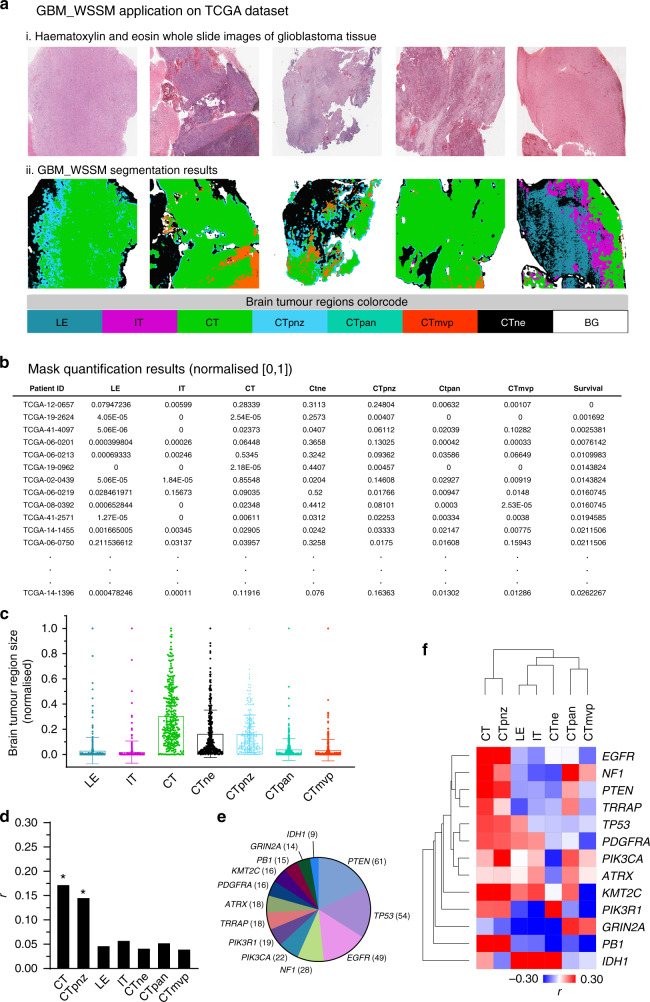
The application of the GBM_WSSM model to the TCGA dataset. **a** Representative results from GBM_WSSM application on the TCGA samples. (i) WSIs from TCGA. (ii) Corresponding masks produced by GBM_WSSM. **b** Measurement of different tumour regions (normalised) and patient survival in each TCGA glioblastoma case. **c** Dot plot graph of the results shown in **b. d** Pearson correlation values between survival rates and normalised areas corresponding to each tumour region (asterisk denotes *p* < 0.05). Comparable statistically significant results were obtained in the presence or absence of outliers using Spearman correlation analysis, which is less sensitive to the presence of outliers. **e** Distribution of glioblastoma patients harbouring specific oncogenic mutations in TCGA. **f** Heatmap plot of Pearson correlation values between the size of the tumour regions and survival rates.

We then applied the GBM_WSSM to the GBM TCGA dataset of whole-slide H&E stained histopathological images. This dataset includes 640 whole-slide images from 329 glioblastomas. All the WSIs were re-sized and fed to the GBM_WSSM as new inputs and their corresponding masks were produced (Fig. 2a), which were also evaluated by our team pathologist for segmentation accuracy. To produce a numerical dataset, the area of the masks for different tumour regions were quantified across all 329 samples analysed. For tumour region quantification, we used the averaged pixel counting approach. In this approach, for each patient, the same pixels related to one region are counted across all masks of that patient and then, the counted value is averaged over the number of slices. This was performed in order to capture the total amount of tumour region across different sections and have a better representation of the volume (measured as total number of pixels) that each of these occupies in the portion of tissue analysed at the same time that normalises the total number of pixels detected for each region by the number of sections available for each patient, as, in many instances, for each patient, there are the different number of available sections. Figure 2b shows the quantified regions for some of the patients. The Grubbs’ test^43^ was then applied on this numerical dataset and 27 outliers were removed in order to obtain a normally distributed survival data, which is required for the analysis of the relationship between segmentation results and patient survival through the calculation of correlation coefficients^44,45^ (Supplementary Table 4).

The results of tumour region size quantification revealed that across the set of TCGA glioblastoma tumours, large areas of WSI images contained brain tumour regions corresponding to the cellular tumour mass including the CT, CTne and CTpnz regions (Fig. 2c, Supplementary Fig. 3, Supplementary Table 4).

This is expected as these regions are the primary focus of the neurosurgery and are thus more likely to appear in resected samples. Other more peripheral regions exhibit higher variability, likely due to differences in surgical procedures and the ability to delimit the tumour boundaries during the operation. With this in mind, we believe our segmentation results agreed with what would be expected from the analysis of data derived from samples collected in multiple centre/institutions, where practices and experimental protocols might not be standardised across the sites and site-to-site differences are likely to be present.

We have previously shown that DCNN models can be trained and applied to TCGA H&E histopathological images to predict brain cancer patient survival^46^ (see also review in ref. ^47^). To analyse the relationship between segmentation results and patient survival, we next performed Pearson correlation analysis between the area of different brain tumour regions and patient survival across (i) all TCGA glioblastomas, and (ii) brain tumours harbouring the 13 most frequent mutations (PTEN, TP53, EGFR, NF1, PIK3CA, PI3KR1, TRAPP, ATRX, PDGFRA, KMT2C, PB1, GRIN2A, IDH1; see Supplementary Fig. 3 for tumour region size distribution across different patient’s cohorts). When we analysed all patients in the TCGA GBM cohort, we found that survival rates are positively correlated with the area CT and CTpnz but poorly correlated with other regions (Fig. 2d). The relatively low correlation values could be due to the heterogeneous nature of the data (i.e. pooled across all patients). To determine if this was the case, we next stratified our analysis by patients harbouring specific oncogenic mutations (Fig. 2e). Mutation-specific stratification resulted in higher correlation values between tumour region size and survival rates and also revealed new additional cases where region size was negatively correlated with survival (Fig. 2f). In brain tumours harbouring mutations in the PIK3R1 and PB1 genes, the presence of LE, IT and CTmvp regions negatively correlated with patient survival, likely reflecting the highly infiltrative nature of tumours bearing these histologies.^48–50^ Moreover, in patients harbouring mutations in the PDGFRA, KMT2C or EGFR genes, we observed a negative correlation between the presence of highly vascularised tumour regions and patient survival, again reflecting the more aggressive phenotype of this type of tumour. Interestingly, we also note that in patients harbouring mutations in the NF1, PTEN, TRAPP, TP53 and PIK3CA, there is a strong positive correlation between survival and central tumour regions (i.e. CT and CTpnz) but little or no correlation with the other infiltrative regions LE, IT and CTmvp). This probably reflects non-invasive and therefore more benign tumours. Overall, these results highlight the varying influence of gene mutations on histopathological features and reveal tumour features associated with prognosis. These data also highlight critical differences that significantly influence the biology of these tumours and their capacity to escape complete resection during surgery as well as to develop therapy resistance.

### Gene signatures of specific brain tumour regions are associated with distinct biological processes in tumour biology and are indicative of patient survival

**Fig. 3:**
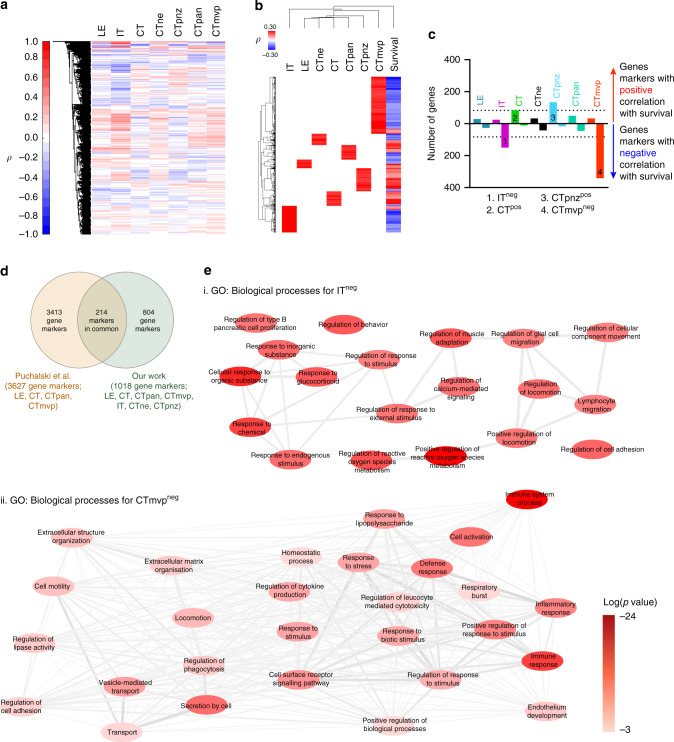
Gene expression analysis in the TCGA segmented tumour regions. **a** Hierarchical cluster analysis (columns only) of Spearman correlation coefficients (ρ) between gene expression and tumour region area measurements for the cohort of TCGA patients in Supplementary Table 1. This cohort was obtained from TCGA cases that have both RNA-seq and WSI data available (See Supplementary Table 4 for Spearman and *p*-values numerical results). **b** Columns for IT, LE, CTne, CT, CTpna, CTpnz and CTmvp are same as in A but the data was filtered for genes that have ρ > 0.1 and this correlation is significantly different from the null hypothesis of no correlation (*p* < 0.05). The ‘survival’ column shows Spearman correlation coefficient values between patient survival and gene expression for all the genes analysed. **c** Bar graph showing the number of markers for each tumour region whose expression is positively and negatively correlated with patient survival (See Supplementary Table 5 for the list of genes for each condition). **d** Venn diagram showing the comparison of gene markers for each tumour region identified in this work and previously by Puchalski et al.^21^ **e**, **f** Summary of GO**:** Biological Processes analysis for IT^neg^ and CTmvp^neg^ gene signatures (see also Supplementary Table 6 for process description, *p*-values and genes associated to each process). The network was simplified by highlighting only GO: Biological Processes with log (*p*-value) < −3.5 and the nodes (i.e. GO: Biological Processes) colour coded (red levels) to highlight the more significant processes associated to each signature. Colour bar indicates different *p*-values for each node.

RNA-seq data from GBM patients in TCGA enabled us to identify gene signatures (markers) associated with each of the different regions. We reason was that genes specific to one region would show higher expression in tumour samples where the area for that region (measured as the total pixel count) is bigger. For this, we used Spearman correlation analysis results between gene expression (>35,000 genes, whose expression was detected in at least 25 patients) and brain tumour area for different brain tumour regions. The results indicated that for different regions, there are both positively and negatively correlated genes (Fig. 3a, Supplementary Table 2). Of these, we focused only on positively (ρ > 0.1) and significantly (*p* < 0.05) correlated genes (Fig. 3b, Supplementary Table 2). Overall, we found that the different brain regions have different numbers of genes that behave as “markers” according to our definitions, with IT and CTmvp regions exhibiting the largest number of marker genes (174 and 373 genes, respectively, Fig. 3c, Supplementary Table 5). Importantly, our analysis validated many of the genes previously annotated as markers in the Ivy GAP Database (Fig. 3d). However, the number of genes identified in the Ivy GAP project was considerably larger than the number that we identified. This is likely due to the fact that not all markers identified in Ivy GAP have a prognostic value as well as the lowest accuracy of Ivy GAP segmentation results, which coupled to analysis of the fewer number of patients, could also lead to a significant increase in the number of false positives.

Next, we analysed the relationship of the region-specific gene signatures with patient survival, which was possible due to the large number of patients whose data are available in the TCGA glioma database (*n* = 129). We found that most of the genes in the CT and CTpnz signatures positively correlated with survival, which agreed with our previous observation in cohorts of patients with a specific set of mutations (Fig. 2d, 3c). We also found that for LE, CTne and CTpan, an approximately equal number of genes positively and negatively correlated with patient survival (Fig. 3c). Interestingly, we found that in IT and CTmvp, the regions with more specific markers, most genes negatively correlated with survival (Fig. 3c). This is consistent with tumour infiltration into the surrounding brain tissue being unfavourable for complete surgical resection and with the capacity for infiltrating tumours to rapidly develop therapy resistance, which together contributes to poor patient survival.

These results revealed different gene signatures for different regions that have different correlations with patient survival. In order to gain insight on these, we then focus on IT and CTmvp gene markers whose expression is negatively correlated with patient survival (IT^neg^ and CTmvp^neg^) as well as CT and CTpnz gene markers whose expression is positively correlated with patient survival (CT^pos^ and CTpnz^pos^). Gene ontology (GO) biological processes analysis allowed us to investigate the cellular processes associated with these gene signatures (Fig. 3e, f and Supplementary Fig. 4, Supplementary Table 6). For the IT^neg^ signature, we identified several GO processes, including cell proliferation in midbrain and regeneration, possibly reflecting the stem-cell-like properties of tumour cells in those regions.^16,19,51^ Processes involving ‘glial cell migration’, ‘locomotion’, ‘cell contractility’, ‘cell movement’ and ‘lymphocyte migration’ were also identified to be associated with the gene signatures specific to this region. This highlights the migratory and invasive potential of cancer cells^52^ as well as the motility of microglia and lymphocytes, suggesting that in the IT region these different cell types within the microenvironment may be cooperating to enhance the invasive capacity of glioblastoma cells.^53^ Interestingly, several GO processes that belong to stress cell responses were also identified in this region (i.e. response to: ‘cycloheximide’; ‘organic’ and ‘inorganic’ substances; ‘glucocorticoids’, and to ‘chemicals’), again reflecting properties of the microenvironment to which tumour and inflammatory cells are exposed.^16^

GO biological processes analysis revealed a more complex microenvironment in the CTmvp^neg^ signature relative to IT^neg^. Important GO processes associated with this signature corresponded to ‘immune’ and ‘immune-inflammatory responses’, ‘cytokine signalling’ and ‘regulation of leukocyte proliferation’, indicating a fine regulation of immune responses in the tumour microenvironment in CTmvp regions. Other key GO processes identified in this region included ‘secretion by cell’, ‘vesicle-mediated transport’, ‘collagen metabolism’, ‘cell motility’, ‘locomotion, ‘extracellular matrix (ECM) organisation’. These are related to the regulation of ECM production and the interaction of that ECM with cells and is consistent with the capacity of tumour cells to interact with the ECM surrounding the vasculature and infiltrate healthy brain tissue along the microvasculature.^54^

In contrast to the IT^neg^ and CTmvp^neg^ signatures, the CT^pos^ and CTpnz^pos^ gene signatures showed a strong connection to those processes related to DNA, RNA and protein synthesis through different metabolic pathways, that align overall with higher macromolecule synthesis rates required by highly proliferative tumour cells (Supplementary Fig. 4). Taken together, these results establish the power of using AI to segment tumour regions in many patient-derived samples and the use of this information for the identification of gene signatures characteristic of each region.

### Gene signatures in different brain regions predict the presence of specific cell types in the tumour microenvironment

The diverse GO processes associated with the different gene signatures motivated us to investigate the identity of the cells that express these signatures in the different tumour regions. Accordingly, we investigated the cell types that are more likely to express these gene signatures in each tumour region using CellKb application software, which uses publicly available scRNA-seq data to predict the cells types that are likely to express a list of differentially expressed genes (“match score”^34^). This match score is calculated for each cell type and correspond to the sum of rank-based scores calculated for overlapping genes between the query and the cell type. The match score thus accounts for the gene rank, the difference in gene ranks and the total number of significant genes in the cell type. Using CellKb, we obtained a list of cell types and for each cell type an associated match score and a list of genes specific for that cell type (Supplementary Table 7).

**Fig. 4:**
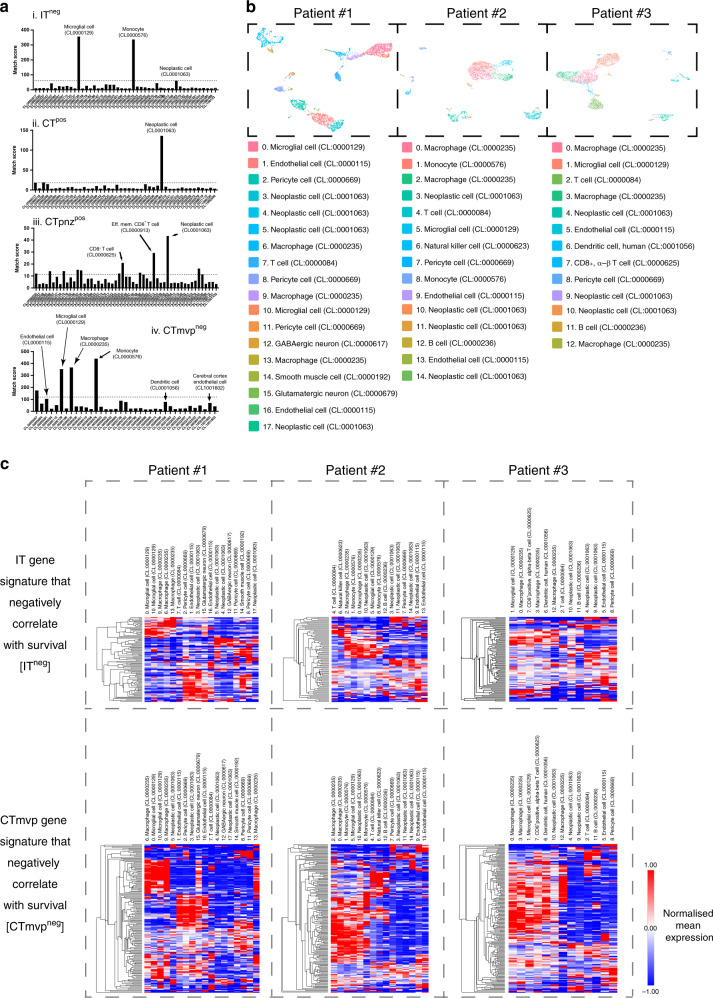
Cell ontology analysis in GBM tumour regions. **a** (i–iv) Cell ontology analysis for the IT^neg^, CT^pos^, CTpnz^pos^ and CTmvp^neg^ gene signatures. The dotted line corresponds to the standard deviation calculated from the average match score across all cell types. **b** Cell type assignment to each of cell clusters from scRNA-seq data derived from three glioblastoma resected tissue samples. **c** Cluster analysis of average gene expression for genes in the IT^neg^ and CTmvp^neg^ signatures in different cell types identified in our scRNA-seq data (See Supplementary Fig. 6 for a high-resolution image that also includes the corresponding gene names).

In this list, a cell type may be predicted more than once, since multiple databases (scRNA-seq experiments) were used. Thus, to uniquely assign a cell type to our gene signature, we weighted the analysis for not only using the cell type match score but also taking into account the number of times a particular cell type was predicted using this analysis (Fig. 4ai–iv, Supplementary Table 8). The results revealed that IT markers that are negatively correlated with survival (IT^neg^) are predicted to be highly expressed by microglial cells (CL:0000129) and monocytes (CL:0000576). This is consistent with the GO processes discussed above but highlighted a key non-cell autonomous role of the tumour microenvironment (microglia) in the poor prognosis of glioblastoma. Cells in CTmvp^neg^ compartment also exhibited monocyte/microglia characteristics. Moreover, this compartment also included cells expressing genes that are specific for macrophages (CL:0000235), endothelial cells (CL:0000115), dendritic cells (CL:0001056), cerebral cortex endothelial cells (CCEC, CL:0001056). Fibroblasts are probably representative of pericytes, which coat blood vessels and contribute to the architecture and function of the blood–brain barrier. The results thus indicate that the genes characteristic of these different regions, that are associated with poor patient prognosis, are more likely expressed by cells of the tumour microenvironment than the cancer cells.

Finally, we performed cell ontology analysis on the CT^pos^ and CTpnz^pos^ regions. These regions are expected to be enriched in tumour cells, which was supported by our analysis revealing that neoplastic cells are more likely to express the gene signature corresponding to each region. Interestingly, T cells were only associated with the CTpnz, a potential inflammatory region that might chemoattract T cells (Fig. 4a). These data also indicate that this region may be particularly important for targeting with checkpoint inhibitor therapy.^55^

CellKb analysis also incorporates scRNA-seq data from a large database of scRNA-seq studies from a range of tissues and disease states that do not necessarily correspond to glioblastoma tumour tissue. We therefore sought to validate our results using scRNA-seq data that we obtained from three glioblastoma patients.^38^ Our previous analysis using cell markers for different cell types in the literature allowed us to define the different cell populations in the scRNA-seq UMAP plots. The tumours analysed were principally composed of glioma-associated macrophages (including microglia and macrophages), lymphocytes, endothelial cells, pericytes and tumour cells.^38^ In order to map the gene signatures identified by semantic segmentation to this data, we first re-analysed our scRNA-seq data using CellKb (Supplementary Table 9). Using this approach, we were able to assign a cell ontology type to each of the different cell clusters in the data derived from each individual patient (Fig. 4b, Supplementary Table 10), which correlated well with our previous manual cell assignment.^38^ Once individual clusters were analysed, we then calculated the average expression of each gene in the gene signatures of the CT^pos^, IT^neg^ and CTmvp^neg^ regions and performed hierarchical clustering to determine which cell types within the brain tumour expresses each of the genes in the different gene signatures that we identified for each region (Fig. 4c, Supplementary Figs. 5 and 6, Supplementary Tables 11–13).

Consistent with our results examining gene expression in publicly available scRNA-seq datasets, we found that the gene signature associated with CT tumour regions is predominantly associated with neoplastic tumour cells across the three patient-derived samples analysed (Supplementary Figs. 5 and 6). Furthermore, a group of genes within this signature was identified to be specifically associated with T, B and NK cells. However, no significant expression of these genes was observed in the microglia/macrophages lineage, which contrasted with what we observed in the corresponding results of the IT and CTmvp regions (Fig. 4c). In these regions, a considerable number of genes of each signature were expressed by cell types including macrophages, microglia and monocytes. A larger number of genes within CTmvp gene signature were also expressed in these cell types compared to that in the IT gene signature. We also observed that pericytes and endothelial cells express a significant number of genes that match the CTmvp signature and a smaller number of genes in the IT signature. Overall, these results reinforce the notion that different brain tumour exhibit different microenvironment properties and cellular compositions that may contribute to prognosis in glioblastoma.

### Gene signatures in different brain regions may mediate tumour-microenvironment interactions

**Fig. 5:**
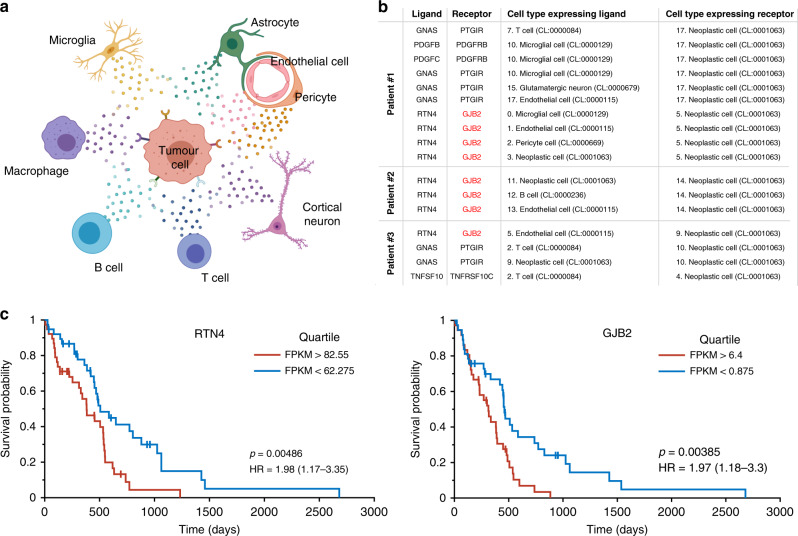
GBM tumour-microenvironment interactions and survival analysis. **a** Schematic representation of different tumour-stroma interactions in glioblastoma. **b** Summary of potential ligand–receptor interactions identified in our dataset. Please note that GNAS (a Gα subunit) and PTGIR (a G protein-coupled receptor) interacts on the cytoplasmic side of the plasma membrane. Supplementary Table 14 provides a full list of ligand–receptor pairs identified in this work. **c** Survival plots of TCGA GBM data for patients with low and high expression of GJB2 and RTN4 reveal their association with a poor prognosis in glioblastoma.

Our cell ontology results indicate that different cell types in the tumour microenvironment contribute to poor patient prognosis. However, the molecular events underpinning this effect are unknown. A possibility is that the gene signatures mediate specific types of cell–cell interactions between tumour cells and the surrounding microenvironment (Fig. 5a). To investigate this, we performed a bioinformatic analysis to explore paracrine ligand–receptor interactions between the previously defined cell clusters in our scRNA-seq data by focusing on ligand–receptor pairs where the tumour cells express the receptor (Fig. 5a). For this, we used the recently developed approach to identify paracrine interactions since it includes one of the most comprehensive datasets for this type of analysis.^40^ After running this bioinformatic pipeline and filtering the results for (i) receptors identified in the IT or CTmvp gene signatures, and;—(ii) expression of the ligand–receptor pair in all three patient-derived scRNA-seq datasets; we identified the reticulon 4 (RTN4): Gap junction beta-2 protein (GJB2) paracrine ligand–receptor pair as a potential mediator of cell–cell interactions between tumour cells and the surrounding microenvironment^56^ (Fig. 5b, Supplementary Table 14). The *RTN4* gene (also known as *NOGO*) encodes three isoforms (isoforms A-C) with Nogo-A enriched in the central nervous system (CNS). NOGO isoforms contain two transmembrane domains and are localised through the exocytic pathway, plasma membrane and cell–cell junctions where it also interacts with cadherin 5 (Cdh5^57^). NOGO proteins have been implicated in glioma cell invasion,^58^ tumour angiogenesis^59^ and regulation of blood vessel homeostasis.^60,61^ In contrast, gap junction protein 2B (GJB2, also known as Connexin26) is a structural component of gap junctions^62–67^ and is expressed at the plasma membranes of cells. GJB2 has previously been identified as a gene associated with prognostic value in brain^68,69^ and pancreatic cancers^70,71^ and contribute to tumorigenesis in breast cancer.^72^ Survival plots of TCGA GBM data for patients with low and high expression of GJB2 and RTN4 reveal their association with a poor prognosis in glioblastoma (Fig. 5c).

## Discussion

Here, we used DCNN for semantic segmentation of different brain tumour regions on TCGA GBM datasets that contain matched histopathological images, patient demographics and gene-expression data. DCNN is a robust tool that can handle the high-level noise in ground truths^41^ derived from semantic segmentation results using machine learning models, that has low-level accuracy.^23^

Using results acquired using DCNN, we have identified specific markers for each of these tumour regions and evaluated their prognostic value in glioblastoma. Moreover, we combined this information with scRNA-seq experiments to determine the cohort of different cell types within these regions that express the genes associated with these signatures as well as protein–protein interactions that mediate tumour-stroma relationships that can serve as potential targets for glioblastoma.

Several studies have been undertaken to characterise gene signatures relating to the tumour microenvironment.^21,73–75^ However, it has not been possible until now to investigate the spatial organisation and possible roles of non-cancer cell types that populate the microenvironment^76^ and pinpoint their occurrence within the context of different brain tumour regions. Amongst, the most notable is the pioneering work by the Allen Institute that lead to the generation of the Ivy GAP database. By using laser microdissection and bulk RNA sequencing, this effort has generated spatial information about the different tumour regions within glioblastomas and associated genetic signatures with them. However, a limitation of this database, is that gene-signature results were derived from laser microdissection of a relatively small number of independent glioblastoma tissue samples (*n* = 32). Although our gene signatures exhibited some overlap with the gene signatures in the reported in the Ivy GAP database, our gene signatures had fewer genes. This is probably a consequence of the thresholds we applied for the correlation coefficient and the *p*-value, to define the different gene signatures and the fact that Ivy GAP tumour regions markers were defined by differences in expression between regions, which is distinct from the correlation versus no correlation approach between gene expression and brain tumour regions taken in this study. Interestingly, our analysis permitted the identification of a large number of genes that did not overlap with the results from Ivy GAP database. This reflects again the power of the combination of AI for segmentation and the possibility of analysing large datasets to be able to perform robust correlation analysis for the identification of gene signatures.

The results of multiple laboratories performing scRNA-seq experiments using cellular suspensions derived from resected tumour tissue^13–17,19,20,38,77^ as well as bioinformatics approaches designed to extract microenvironment features from bulk RNA-seq data in TCGA^78^ have significantly advanced the characterisation of the genetic signatures and cellular composition of the tumour microenvironment in glioblastoma. These previous studies have provided significant insight into the tumour cell heterogeneity in glioblastoma as well as the cellular composition of the tumour microenvironment.^79^ Moreover, these experiments have not only provided a better understanding of tumour plasticity but also the transcriptional program changes that occur in different cell types in the tumour microenvironment.^77^ However, it has not been possible until now to investigate the spatial organisation of non-cancer cell types that populate the microenvironment and pinpoint their occurrence within the context of different brain tumour regions. In a recent study, Rajapakse et al. used computational modelling to define cancer cell states and allocate active and dormant cancer cell types within these different tumour regions.^80^ In contrast, in this work, we combined publicly available scRNA-seq data (using the CellKb platform) and our own experiments to determine the gene signatures expressed by different cell types in the microenvironment and how these relate to different tumour compartments. Although spatial transcriptomics^81–83^ and multiplex immunofluorescence imaging^74,75^ approaches can offer similar outcomes to that presented here, these techniques are still prohibitively expensive and time-consuming. We expect these results to facilitate the spatial analysis of tumour biopsies and promote the adoption of complementary techniques such as spatial transcriptomics^81–83^ and highly multiplexed immunofluorescence imaging.^74,75^ Although expensive and time-consuming, these approaches enable high-resolution detection of RNA or protein in tissue sections, which facilitates precise spatial analysis of the tumour microenvironment and measurements of distances (and possible interactions) between different cells of interest when combined with single-cell RNA sequencing analysis.^84^

scRNA-seq data have also proved to be a powerful tool for assessing potential protein–protein interactions that can control tumour cell behaviour.^40,85^ Using these methods, we investigated membrane receptors expressed on tumour cells that can interact with secreted and/or membrane proteins, and determined whether these ligand–receptor interactions are part of the gene signatures that we have identified. Interestingly, we found the GJB2 (also known as connexin 26): RTN4 (also known as NOGO) pair in the CTmvp signature, with both genes being indicative of poor prognosis in glioblastoma.

Although the role of connexins has been primarily associated with its ability to function as hemichannels forming a direct transmembrane communication pathway between neighbouring cells, recent studies of clinical samples suggested connexins can contribute to cancer progression through multiple pathways, namely (1) gap junction intercellular communication, (2) C-terminal tail-mediated signalling and (3) cell–cell adhesion during gap junction formation.^86^ In our data, the expression of GJB2 (and another receptor for RTN4, RTN4R) was restricted mostly to cancer cells, whereas the expression of RTN4 was more widely distributed between cells in the tumour microenvironment including microglial cells, endothelial cells, pericytes and B cells. GJB2 is a tetraspan membrane protein with two extracellular domains and is expressed in the plasma membrane. GJB2 has previously been identified as a gene with prognostic significance in brain,^68,69^ pancreatic^70,71^ and breast cancer.^72^ RTN4 has two long (32 aa) transmembrane domains and the region between these domains (NOGO-66) has been shown to mediate protein interaction with cells expressing RTN4 receptors.^87,88^ The long transmembrane domains have been also postulated to confer distinct topological organisations of this protein, particularly exposing the N-terminus (the more divergent domain between RTN proteins) extracellularly.

In terms of downstream signalling, RTN4 localises both at the endoplasmic reticulum and at the plasma membrane. In the endoplasmic reticulum, it is known to interact with and suppress the anti-apoptotic functions of Bcl2 and BclXL,^89^ suggesting a role as a tumour suppressor. However, more recent data in prostate cancer has shown that RTN4 regulates cell fate and its overexpression led to cell cycle arrest and senescence.^90^ Besides, RTN4 binding to RTN4R, the more established receptor RTN4, contributes to ROCK and STAT3 activation in the cell expressing RTN4R,^88^ which can contribute to glioblastoma tumour cells invasion (through ROCK^91^) and chronic microglia activation (through ROCK and STAT3^92,93^). It is still unclear how RNT4’s interaction with GJB2 and RTN4R contributes to poor prognosis in glioblastoma, but redistributing RTN4 from the ER to the plasma membrane and preventing it to interact with other RTN4 intracellular binding partners could be a possible therapeutic strategy that needs further investigation.

In summary, our analysis of the TCGA GBM database using segmentation of histopathological images provides new opportunities for the characterisation of the tumour microenvironment in glioblastoma within distinctly spatial regions within these very heterogeneous brain tumours. These results have led to the identification of novel prognosis-associated region-specific gene signatures and targets for treating glioblastoma and are now available to the rest of the brain cancer research community.

## Supplementary information

Supplementary material

Supplementary legends

## Data Availability

Computational source codes along with the datasets used for image segmentation and RNA-seq analysis are provided in the Supplementary Information and have been deposited at GitHub (https://github.com/amin20/GBM_WSSM).
