# Supplementary material for: A deep convolutional neural network for segmentation of whole-slide pathology images identifies novel tumour cell-perivascular niche interactions that are associated with poor survival in glioblastoma
Source: Br J Cancer. 2021 Apr 29;125(3):337–50. doi: 10.1038/s41416-021-01394-x (PMC8329064; doi:10.1038/s41416-021-01394-x)
Supplement: Supplementary file 2 — Supplementary legends [file 41416_2021_1394_MOESM2_ESM.pdf]

List and captions for Supplementary Figures, Tables and Materials, including Figures

A deep convolutional neural network for segmentation of whole-slide pathology images identifies novel tumour cell-perivascular niche interactions that are associated with poor survival in glioblastoma.

Amin Zadeh Shirazi[1,2+*], Mark D. McDonnell[2+], Eric Fornaciari[3+], Narjes Sadat Bagherian[4], Kaitlin G. Scheer[1], Michael S. Samuel[1,8], Mahdi Yaghoobi[5], Rebecca J. Ormsby[6], Santosh Poonnoose[6,7], Damon J. Tumes[1], and Guillermo A. Gomez[1+*].

[1] Centre for Cancer Biology, SA Pathology and University of South Australia, Adelaide, Australia

[2] Computational Learning Systems Laboratory, UniSA STEM, University of South Australia, Mawson Lakes, S.A., 5095, Australia

[3] Department of Mathematics of Computation, University of California, Los Angeles (UCLA), USA

[4] Mashhad University of Medical Sciences, Mashhad, Iran

[5] Electrical and Computer Engineering Department, Department of Artificial Intelligence, Islamic Azad University, Mashhad Branch, Mashhad, Iran

[6] Flinders Health and Medical Research Institute, College of Medicine & Public Health, Flinders University, Adelaide, Australia

[7] Department of Neurosurgery, Flinders Medical Centre, Bedford Park, Australia

[8] Adelaide Medical School, University of Adelaide, Australia

Supplementary Figure 1. A, Flowchart diagram for the implementation of a DCNN model for semantic segmentation of glioblastoma histopathological images. **B**, Examples of outliers in the IVY-GAP histopathological image database. These outliers were identified by our pathologist and removed before this database was used for training of different DCNN models. The figure shows histopathological images and the corresponding ground truth (GT) images obtained from the IVY-GAP portal. As can be seen in the GT images, the original method used for segmenting the histopathological images is not entirely accurate and significant parts of the images are missing information or were poorly segmented. For example, the first and second GTs have missed the necessary information related to the original slides (i.e. the GT images were "cropped" on some regions). Also, in the third and fourth examples, there are at least three different regions including

Cellular Tumour (CT), Infiltrating Tumour (IT), and in some parts, Cellular Tumour necrosis (CTne), but the only region detected in their corresponding GTs is the over-segmented Cellular Tumour (CT) region (shown by the green colour). As the images were thus not of sufficient quality to be used for the training of our DCNN models, they were considered to be outliers and were removed to protect the integrity of the training dataset.

Supplementary Figure 2. A, p-values density distribution for Spearman correlation coefficients between gene expression and brain tumour region (LE, IT, CT, CTne, CTpnz, CTpan and CTmvp) calculated for all genes listed in Supplementary Table 4. B, p-values density distribution for Spearman correlation coefficients between gene expression and patient survival (survivals rates are listed Supplementary Table 3). C.i, Gene expression correlation analysis for all protein coding genes across all patient analysed. C.ii, Brain tumour region size correlation analysis for all tumour regions across all patients analysed. C.iii, Gene expression correlation analysis for all protein coding genes identified as "gene marker" and whose expression was also detected in our scRNAseq dataset.

Supplementary Figure 3. Tumour region size (in pixels) distribution for all TCGA patients or TCGA patients harbouring specific mutations in the PTEN, TP53, EGFR, NF1, PIK3R1, RB1, ATRX, PIK3CA, TRRAP, KMT2C and GRIN2A genes.

Supplementary Figure 4. Summary of GO: Biological Processes analysis for CTpos (i) and CTpnzpos (ii) gene signatures (see also Supplementary Table 6 for process description, p-values and genes associated with each process). The network was simplified by highlighting only GO: Biological Processes with log (p-value)<-3.5 and the nodes (i.e. GO: Biological Processes) color coded (red levels) to highlight the more significant processes associated to each signature. Color bar indicates different p values for each node.

Supplementary Figure 5. Cluster analysis of average gene expression for genes in the CT^{pos} signatures in different cell types identified in our scRNA-seq data (See Supplementary Figure 6 for a high-resolution image that also includes the corresponding gene names).

Supplementary Figure 6. Cluster analysis of average gene expression for genes in the ITneg, $CTmvp^{neg}$ and CT^{pos} signatures in different cell types and across all patients in our scRNA-seq data.

Supplementary Table 1. Glioblastoma TCGA cases that were used for correlation analysis. The table also contains information related to biospecimen data for RNA and slide matched samples (case ID, sample Submitter ID, tissue portion (Portion ID) as well as the data filename in the TCGA portal for access to the file with the corresponding RNA expression data.

Supplementary Table 2. Spearman correlation coefficients (and corresponding p-values) between gene expression and brain tumour region area.

Supplementary Table 3. The details of different experiments including re-sized image, patch size extracted, and hyperparameter tuning based on the random search approach to obtain the best model for semantic segmentation of GBM WSIs. The best experiment has been bolded in #47.

Supplementary Table 4. Brain tumour regions size quantification based on the areas segmented by applying the GBM_WSSM model to WSIs for each patient from TCGA dataset (329 total GBM patients). The left part indicates the original values measured and the right part shows the values normalized in the range [0, 1].

Supplementary Table 5. Gene list for gene signatures corresponding to different brain tumour regions and its correlation (positive or negative) with patient survival.

Supplementary Table 6. GO: biological processes results including description, p-values and gene lists for CT^{pos}, $CTpnz^{pos}$, IT^{neg} and $CTmvp^{neg}$ gene signatures.

Supplementary Table 7. CellKb raw data results for IT^{neg}, CT^{pos}, $CTpnz^{pos}$ and $CTmvp^{neg}$ gene signatures.

Supplementary Table 8. Results from processing CellKb raw data to uniquely assign a score to a cell type. This score is obtained by adding match scores for cell types that appear multiple times in the CellKb raw data (i.e. as shown in Supplementary Table 7).

Supplementary Table 9. Results from processing CellKb raw data derived from the analysis of differentially expressed genes in our scRNA-seq data.

Supplementary Table 10. Results from processing CellKb raw data derived from the analysis of differentially expressed genes in our scRNA-seq data. This table only includes the cell type with the highest score for each scRNA-seq cluster, which then is used to label the cluster. It was noted that in some opportunities Fibroblasts appears as the cell type with the highest score for some scRNA-seq clusters. However, fibroblast in the brain is expected to be a very rear cell type. Because of their similitudes with pericytes and because pericytes are in these cases ranked 2nd, these clusters were assigned as pericytes in Figure 4.

Supplementary Table 11. Normalized, average gene expression (CT^{pos} signature) per cell type across the three patient-derived scRNA-seq datasets.

Supplementary Table 12. Normalized, average gene expression (IT^{neg} signature) per cell type across the three patient-derived scRNA-seq datasets.

Supplementary Table 13. Normalized, average gene expression ($CTmvp^{neg}$ signature) per cell type across the three patient-derived scRNA-seq datasets.

Supplementary Table 14. List of ligand-receptor interaction pairs and LR score obtained from scRNA-seq analysis of resected glioblastoma tissue sample (patients #1-#3) using SingleCellSignalR.

Supplementary Material 1. Matlab code for Spearman correlation analysis calculation between tumour region area and gene expression.

Supplementary Material 2. Matlab code to to import TCGA RNAseq data.

Supplementary Material 3. Matlab code to import tumour region segmentation data.

Supplementary Figure 1

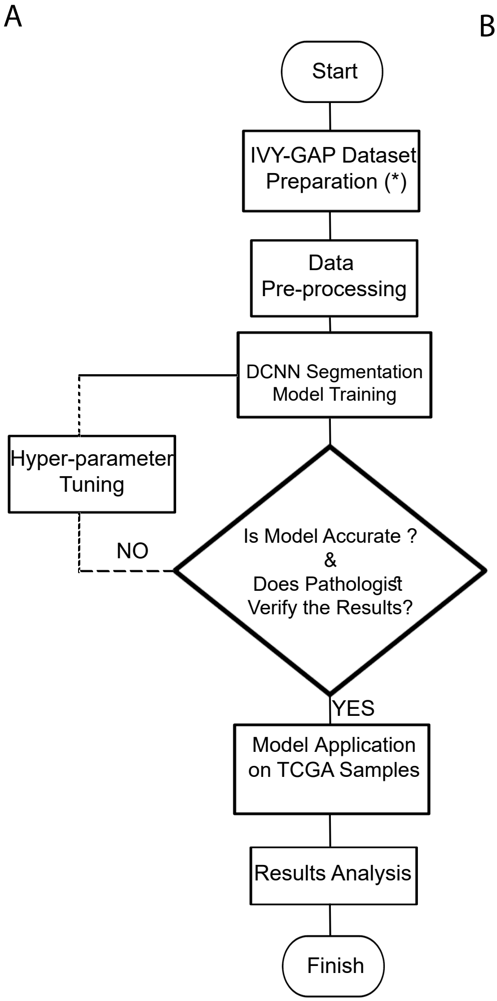

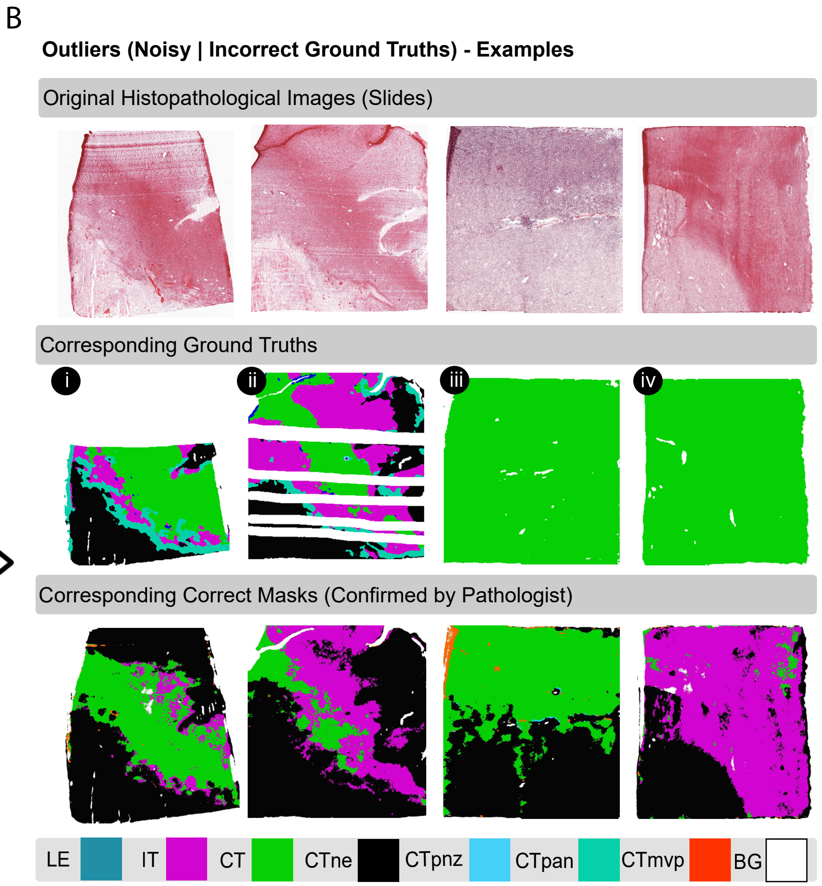

Supplementary Figure 2

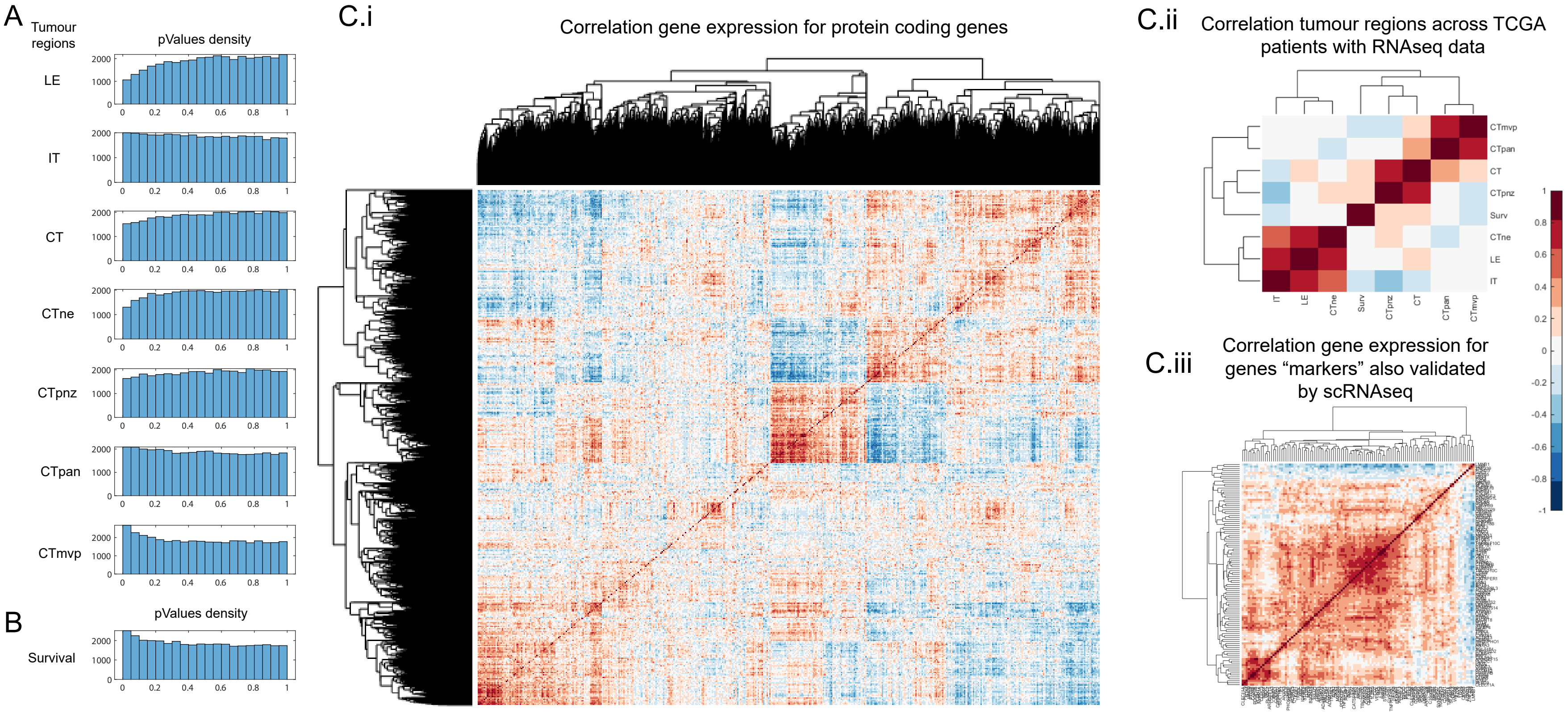

Supplementary Figure 3

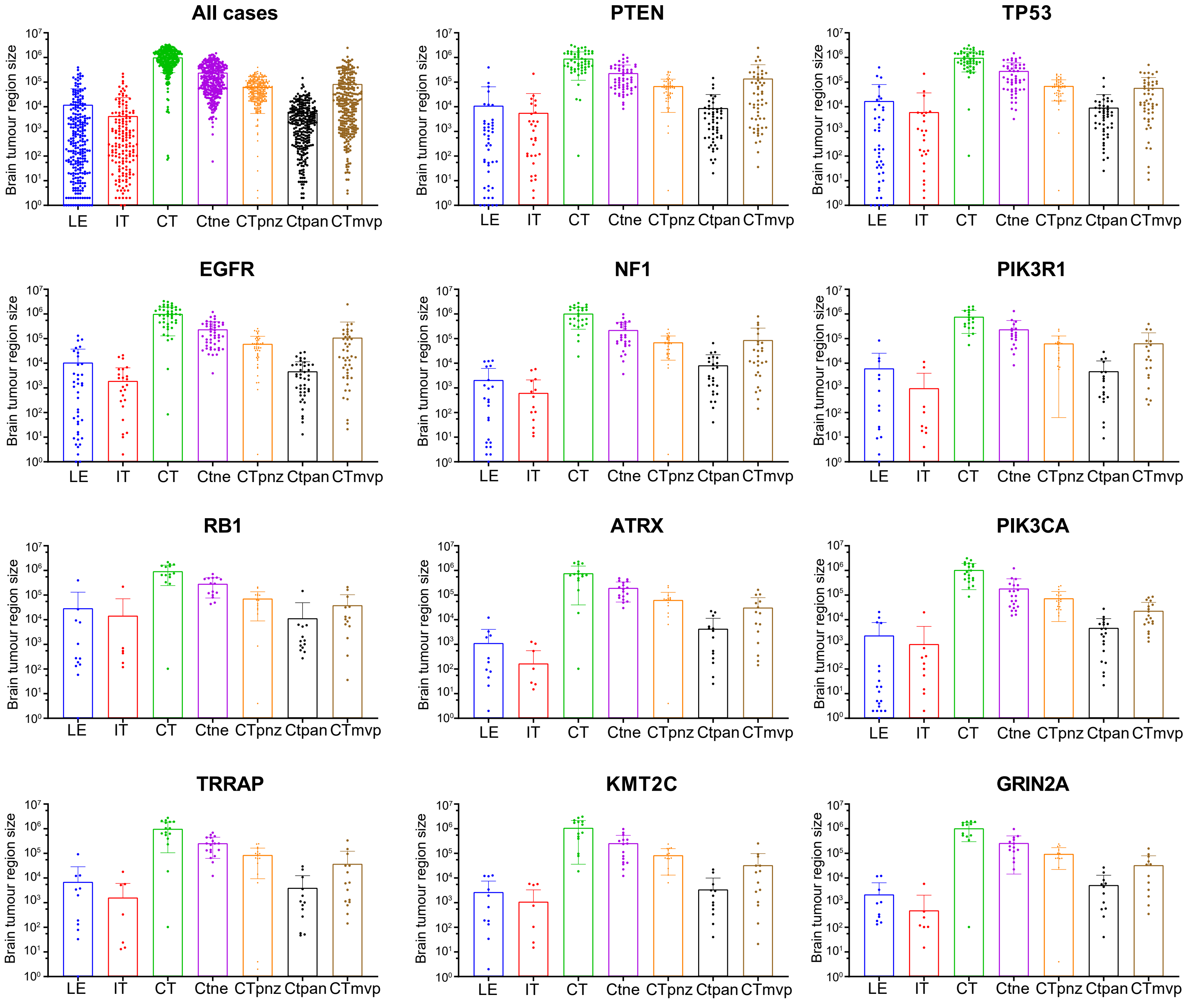

Supplementary Figure 4

i. GO: Biological Processes for CT^{pos}

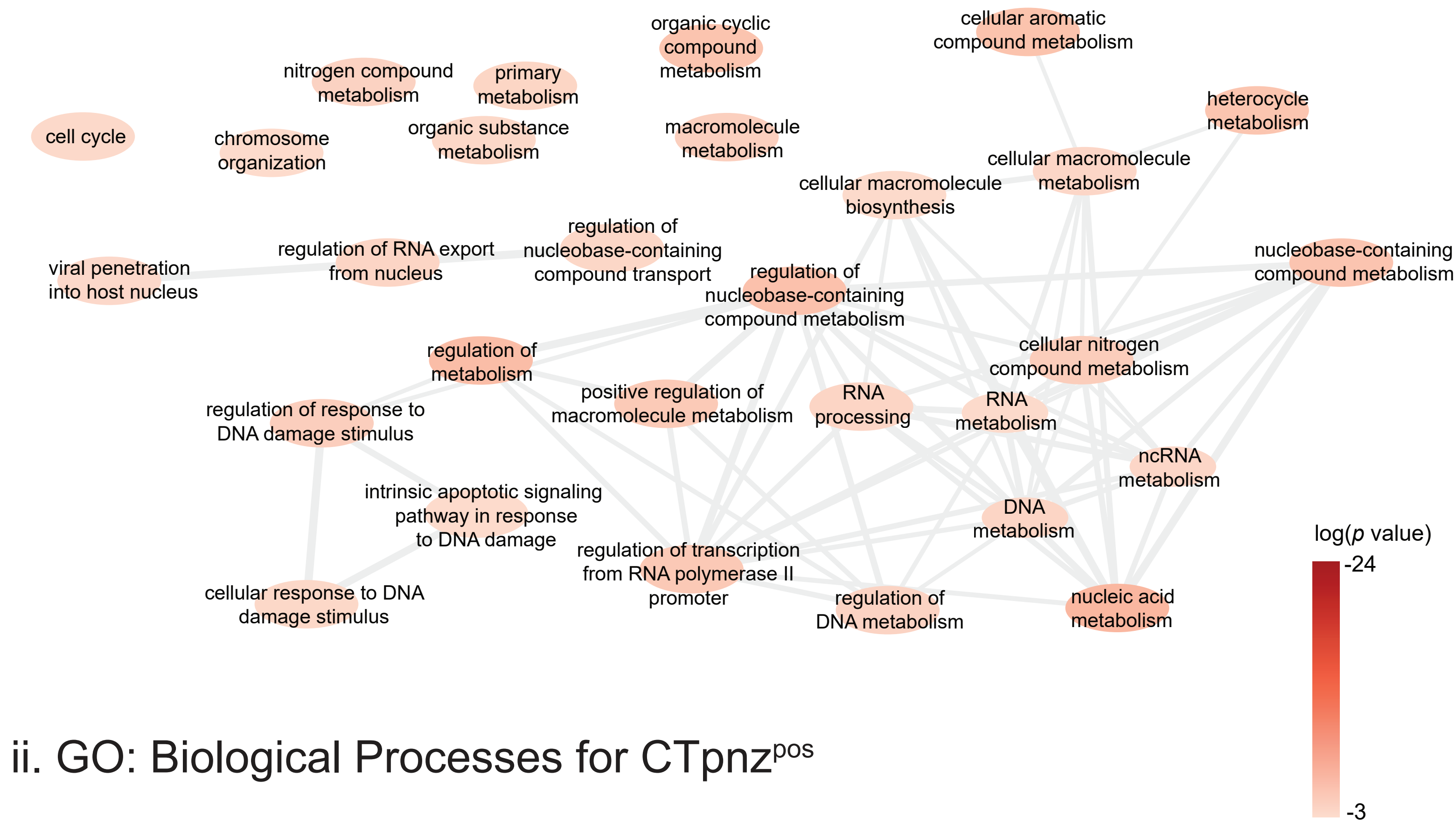

ii. GO: Biological Processes for CTpnz^{pos}

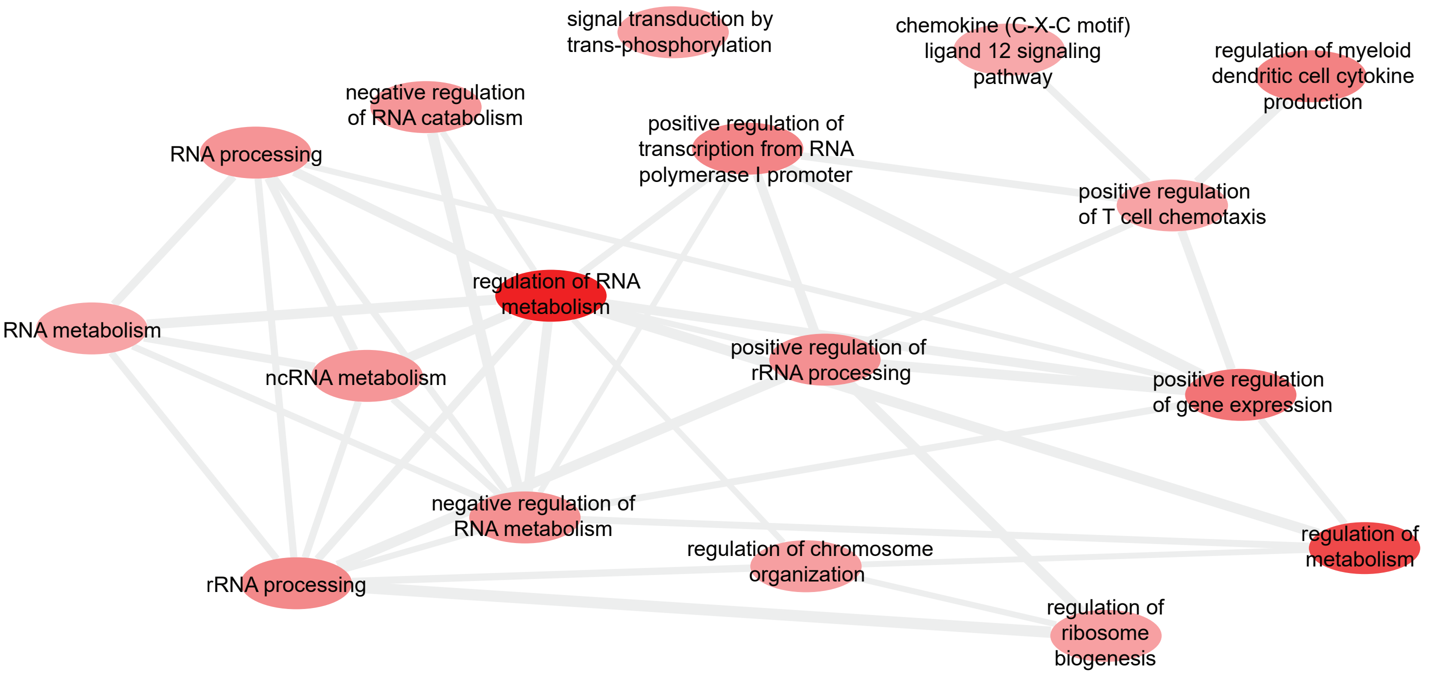

Supplementary Figure 5

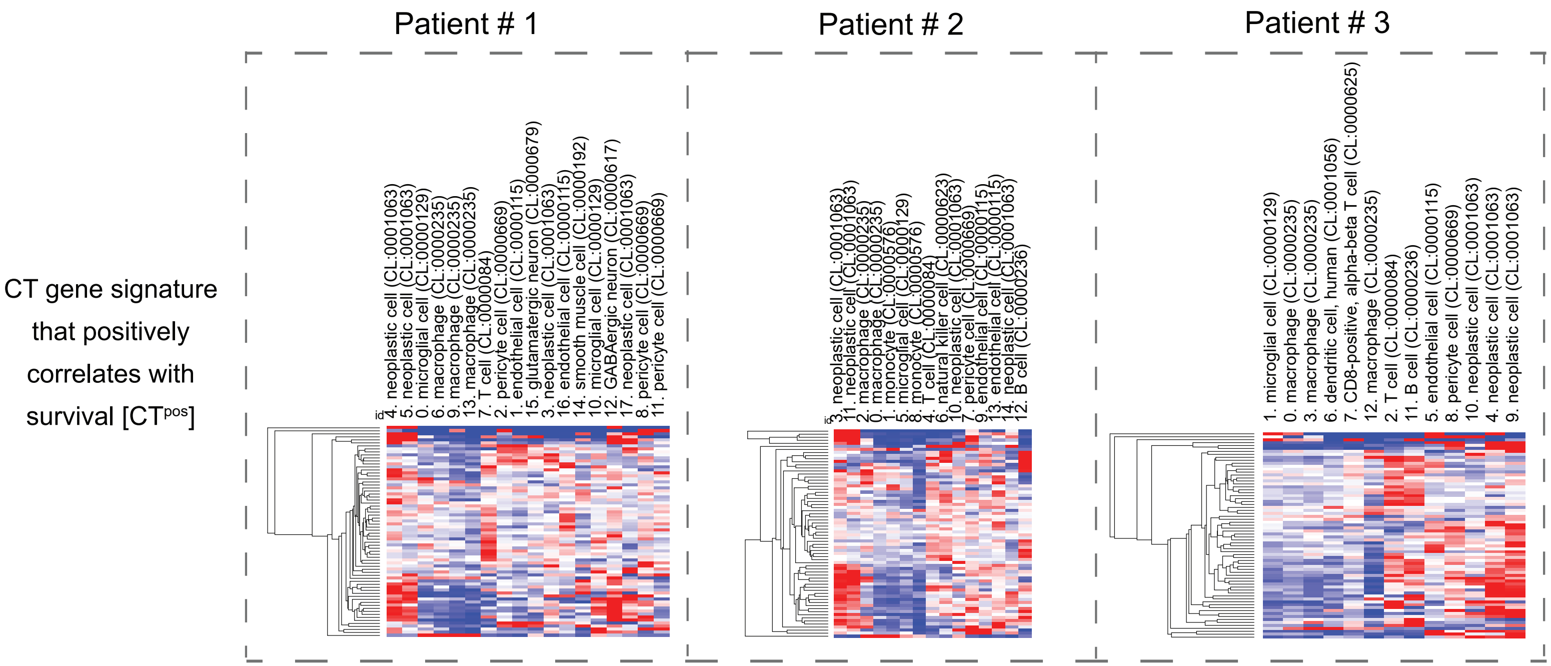

Supplementary Figure 6

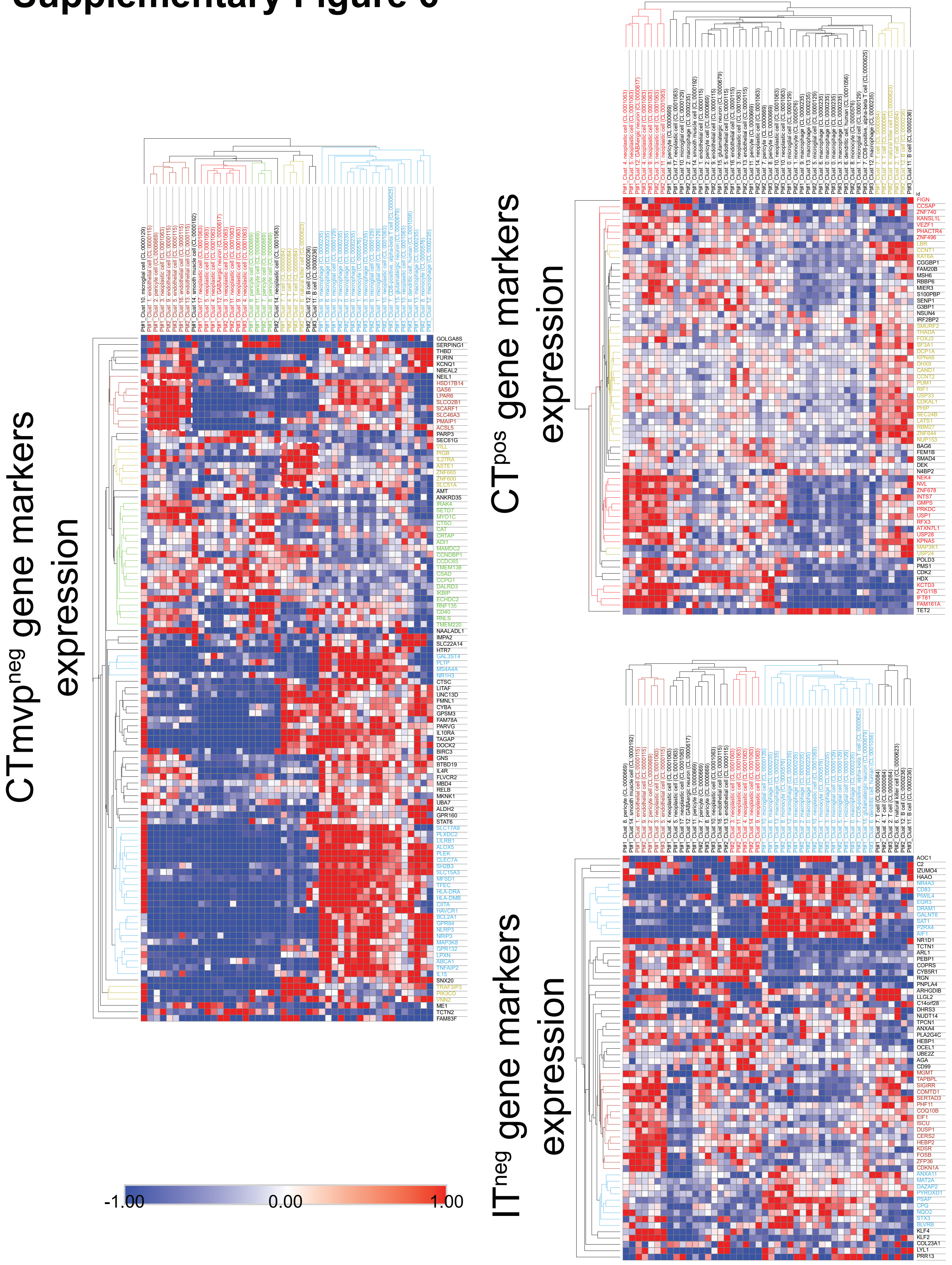

Supplementary Material 1

```
run Import_Segmentation_Derived_Data_V3.m
'Import segmentation data: completed'
run Import_RNAseq_Data_V3.m
'Import RNAseq data: completed'

%% Filter thresholds
Minimal_number_patients_with_expression_of_a_given_gene=25;
Threshold_pValue=0.05;
Threshold_rho=0.1;
%% convert to double input data and convert 0 to NaN
Segmentation_DATA=str2double(Segmentation_Derived_Data(:,2:9));
Segmentation_DATA(Segmentation_DATA==0)=NaN;
Gene_expression_unique(Gene_expression_unique == 0) = NaN;
sizesegmentation_DATA=size(Segmentation_DATA);
Total_number_of_patients=sizesegmentation_DATA(1);

GeneExpression_DATA=Gene_expression_unique;
y_values=Gene_Names;


x_values=Column_Names_Segmentation_Derived_Data(2:9);
x_values2=["LE", "IT", "CT", "CTne", "CTpnz", "CTpan", "CTmvp","Surv"];
save('Data_for_correlation_Unfiltered.mat','x_values','x_values2','y_values','GeneExpression_DATA','Segmentation_DATA','Total_number_of_patients','Minimal_number_patients_with_expression_of_a_given_gene','Threshold_pValue','Threshold_rho')
;

%% Filter 1, remove rows of genes whose expression is only reported in <25
of cases.
clear
load('Data_for_correlation_Unfiltered.mat');

sizeGeneExpression_DATA=size(GeneExpression_DATA);
Total_number_of_genes=sizeGeneExpression_DATA(1);

k=0;
for i=1:Total_number_of_genes;
       A=GeneExpression_DATA(i,:);
       number_of_patients_expressing_gene=Total_number_of_patients-
sum(sum(double(isnan(A))));
    if number_of_patients_expressing_geneThreshold_rho);
rhoMASK(:,8)=1;%here add a one
pval(:,1:8)=pval(:,1:8).*double(rhoMASK(:,1:8));
rho(:,1:8)=rho(:,1:8).*double(rhoMASK(:,1:8));

rho_constant_rows=sum(rho(:,1:7)')';
sizeGeneExpression_DATA=size(GeneExpression_DATA);
Total_number_of_genes=sizeGeneExpression_DATA(1);
k=0;
for i=1:Total_number_of_genes;

    if rho_constant_rows(i)==0;
        k=k+1
        rows_to_delete(k,1)=i;
    end
end



y_values(rows_to_delete,:)=[];
rho(rows_to_delete,:)=[];
pval(rows_to_delete,:)=[];
GeneExpression_DATA(rows_to_delete,:)=[];

sizeGeneExpression_DATA=size(GeneExpression_DATA);
Total_number_of_genes=sizeGeneExpression_DATA(1);

save('Correlation_Data_Filter3.mat','rho','pval','x_values','x_values2','y_values','GeneExpression_DATA','Segmentation_DATA','Total_number_of_patients','Minimal_number_patients_with_expression_of_a_given_gene','Threshold_pValue','Threshold_rho')
;



'Filter 3: completed'



%% filter 4 to isolate genes that belongs to one "tumour region" only
clear
load('Correlation_Data_Filter3.mat');

DEG_MASK(:,1:7)=double(rho(:,1:7)~=0);
rows_to_delete=sum(DEG_MASK(:,1:7)')';
rows_to_delete=(rows_to_delete>1);


y_values(rows_to_delete,:)=[];
rho(rows_to_delete,:)=[];
pval(rows_to_delete,:)=[];
GeneExpression_DATA(rows_to_delete,:)=[];

sizeGeneExpression_DATA=size(GeneExpression_DATA);
Total_number_of_genes=sizeGeneExpression_DATA(1);


save('Correlation_Data_Filter4.mat','x_values','x_values2','y_values','GeneExpression_DATA','Segmentation_DATA','rho','pval','Total_number_of_genes','Total_number_of_patients','Minimal_number_patients_with_expression_of_a_given_gene','Threshold_pValue','Threshold_rho')
;

'Filter 4: completed'
%%
'Filter 5: keep those genes that corresponds to protein coding genes'
clear
load('Correlation_Data_Filter4.mat');

clases_to_keep='protein_coding';
run Import_ENSG_names_BioMart_Data_V1.m

size_genes_to_keep=size(ENSGlistAllTCGAgenesBioMart);

number_of_genes_to_keep=size_genes_to_keep(1);
k=0;

for i=1:Total_number_of_genes;

    for j=1:number_of_genes_to_keep;

    if extractBefore(y_values(i),".")==ENSGlistAllTCGAgenesBioMart(j,1);
        k=k+1;
        gene_list(k,1)=y_values(i);
        gene_list(k,2)=ENSGlistAllTCGAgenesBioMart(j,4);
        GeneExpression_DATA_final(k,:)=GeneExpression_DATA(i,:);
        rho_final(k,:)=rho(i,:);
        pval_final(k,:)=pval(i,:);

    end

    end

end

clear('y_values','rho','pval','GeneExpression_DATA','genes_to_keep');

y_values=gene_list(:,1);
y_values_gene_names=gene_list(:,2);
rho=rho_final;
pval=pval_final;
GeneExpression_DATA=GeneExpression_DATA_final;

sizeGeneExpression_DATA=size(GeneExpression_DATA);
Total_number_of_genes=sizeGeneExpression_DATA(1);


save('Correlation_Data_Filter5.mat','x_values','x_values2','y_values','y_values_gene_names','GeneExpression_DATA','Segmentation_DATA','rho','pval','Total_number_of_genes','Total_number_of_patients','Minimal_number_patients_with_expression_of_a_given_gene','Threshold_pValue','Threshold_rho')
;

'Filter 5: completed'

%%
'Filter 6: Create a threshold for correlation of survival data'

clear
load('Correlation_Data_Filter5.mat')

rows_to_delete=abs(rho(:,8))
```

Supplementary Material 2

```
%% Import data from spreadsheet containing the matrix between Cases ID and
Gene Expression
%    Workbook: C:\Users\gomezga\OneDrive - University of South
Australia\Amin's Project\Amin_project_RNAseq_DATA_Analysis.xlsx
%    Worksheet: Files_RNAseq_TCGA_
%% Setup the Import Options and import the data
opts = spreadsheetImportOptions("NumVariables", 4);
% Specify sheet and range
opts.Sheet = "DATAfromRNAseq";
opts.DataRange = "A2:D87";
% Specify column names and types
opts.VariableNames = ["CaseID", "FileName", "SampleID", "SampleType"];
opts.VariableTypes = ["string", "string", "string", "string"];
% Specify variable properties
opts = setvaropts(opts, ["CaseID", "FileName", "SampleID", "SampleType"],
"WhitespaceRule", "preserve");
opts = setvaropts(opts, ["CaseID", "FileName", "SampleID", "SampleType"],
"EmptyFieldRule", "auto");
% Import the data
Tbl_RNAseq_txtFile_CaseID = readmatrix("C:\Users\gomezga\OneDrive -
University of South Australia\Amin's
Project\Amin_project_RNAseq_DATA_Analysis.xlsx", opts, "UseExcel", false);

% Clear temporary variables
clear opts
clear ('numIdx');

%% FOR LOOP TO IMPORT THE FIRST COLLUMN OF ALL FILES AND CHECK THEY ARE
CORRECT AS WELL AS IMPORT TXT FILES WITH EXPRESSION DATA
size_Tbl_RNAseq_txtFile_CaseID=size(Tbl_RNAseq_txtFile_CaseID);
number_of_files=size_Tbl_RNAseq_txtFile_CaseID(1);

for i=1:number_of_files;

opts = delimitedTextImportOptions("NumVariables", 2);
% Specify range and delimiter
opts.DataLines = [1, Inf];
opts.Delimiter = "\t";
% Specify column names and types
%opts.VariableNames = ["ENSG000002422682", "VarName2"];
opts.VariableTypes = ["string", "string"];
% Specify file level properties
opts.ExtraColumnsRule = "ignore";
opts.EmptyLineRule = "read";
% Specify variable properties
opts = setvaropts(opts, "WhitespaceRule", "preserve");
opts = setvaropts(opts, "EmptyFieldRule", "auto");

filename=Tbl_RNAseq_txtFile_CaseID(i,2);
filename=convertStringsToChars(filename);
filename=filename(1:end-3);
filename = convertCharsToStrings(filename);

path_filename=strcat("C:\Users\gomezga\OneDrive - University of South
Australia\Amin's Project\RNAseq_DATA\Uncompressed\",filename);

txt_File = readmatrix(path_filename, opts);
%the below tables contain the imported data
Gene_access_names(:,i)=txt_File(:,1);
Gene_expression(:,i)=txt_File(:,2);
end


Gene_expression=str2double(Gene_expression);
size_Gene_access_names=size(Gene_access_names);
number_genes=size_Gene_access_names(1);

for i=1:number_genes;
A(i,1)=Gene_access_names(i,1);
B(i,1)=sum(sum(double((A(i)==Gene_access_names(i,:)))));
end
C=sum(double(B(1:number_genes)~=number_of_files));

if C~=0
    'genes are not ordered properly across the samples -i.e. rows (gene
name) do not match between diferent CASE_IDs'
end

Gene_Names=Gene_access_names(:,1);

% Clear temporary variables
clear opts
clear
('filename','A','B','C','pat_filename','size_Gene_access_names','txt_File','Gene_access_names','path_filename')
;


%% For LOOP to remove replicates in the table of CASE_ID and RNAseq_DATA

unique_CASE_IDs = unique(Tbl_RNAseq_txtFile_CaseID(:,1));
size_unique_CASE_IDs=size(unique_CASE_IDs);
number_of_unique_cases=size_unique_CASE_IDs(1);


k=0;
for i=1:number_of_unique_cases;
       unique_CASE_ID=unique_CASE_IDs(i);
       identical_cases=0;

       for j=1:number_of_files;
          if Tbl_RNAseq_txtFile_CaseID(j,1)==unique_CASE_ID;
             identical_cases=identical_cases+1;
             if identical_cases>1;
                 k=k+1;
                 rows_to_delete(k)=j;

             end
          end
       end
end
rows_to_delete=rows_to_delete';

Tbl_RNAseq_txtFile_CaseID_unique=Tbl_RNAseq_txtFile_CaseID;
Tbl_RNAseq_txtFile_CaseID_unique(rows_to_delete,:)=[];
%% For LOOP to average replicates in the table of CASE_ID and RNAseq_DATA
unique_CASE_IDs = unique(Tbl_RNAseq_txtFile_CaseID(:,1));
size_unique_CASE_IDs=size(unique_CASE_IDs);
number_of_unique_cases=size_unique_CASE_IDs(1);
```

```
for i=1:number_of_unique_cases;
       unique_CASE_ID=unique_CASE_IDs(i);
       k=0;

       for j=1:number_of_files;
          if Tbl_RNAseq_txtFile_CaseID(j,1)==unique_CASE_ID;
             k=k+1;
              rows_to_average(i,k)=j;
          end
       end
  if k==1;

Gene_expression_unique(:,i)=Gene_expression(:,nonzeros(rows_to_average(i,:)))
;
  elseif k>1

Gene_expression_unique(:,i)=mean(Gene_expression(:,nonzeros(rows_to_average(i,:)))')'
;
  end


end

%clear temporary variables
clear('unique_CASE_IDs',
'size_unique_CASE_IDs','number_of_unique_cases','identical_cases','i','j','k','rows_to_average','rows_to_delete','size_Tbl_RNAseq_txtFile_CaseID','unique_CASE_ID'
)
```

Supplementary Material 3

```
%% Import data from spreadsheet
% Script for importing data from the following spreadsheet:
%
%    Workbook: C:\Users\gomezga\OneDrive - University of South
Australia\Amin's Project\Amin_project_RNAseq_DATA_Analysis.xlsx
%    Worksheet: DATA_from_MASKs
%
% Auto-generated by MATLAB on 14-Jul-2020 13:37:03

%% Setup the Import Options and import the data
opts = spreadsheetImportOptions("NumVariables", 9);

% Specify sheet and range
opts.Sheet = "DATA_from_MASKs";
opts.DataRange = "A2:I81";

% Specify column names and types
opts.VariableNames = ["TCGA_IDs", "LE_Teal_or_Blue", "IT_Purple",
"CT_Green", "CTne_Black", "CTpnz_Light_Blue", "CTpan_Sea_Green",
"CTmvp_Red", "SR_Days_N"];
opts.VariableTypes = ["string", "string", "string", "string", "string",
"string", "string", "string", "string"];

% Specify variable properties
opts = setvaropts(opts, ["TCGA_IDs", "LE_Teal_or_Blue", "IT_Purple",
"CT_Green", "CTne_Black", "CTpnz_Light_Blue", "CTpan_Sea_Green",
"CTmvp_Red", "SR_Days_N"], "WhitespaceRule", "preserve");
opts = setvaropts(opts, ["TCGA_IDs", "LE_Teal_or_Blue", "IT_Purple",
"CT_Green", "CTne_Black", "CTpnz_Light_Blue", "CTpan_Sea_Green",
"CTmvp_Red", "SR_Days_N"], "EmptyFieldRule", "auto");

% Import the data
Segmentation_Derived_Data = readmatrix("C:\Users\gomezga\OneDrive -
University of South Australia\Amin's
Project\Amin_project_RNAseq_DATA_Analysis.xlsx", opts, "UseExcel", false);
Column_Names_Segmentation_Derived_Data=opts.VariableNames;

%% Clear temporary variables
clear opts
```
